# Editorial: Decoding novel therapeutic targets, biomarkers, and drug development strategies against neurodegenerative disorders—A multi-omics approach

**DOI:** 10.3389/fncom.2023.1227850

**Published:** 2023-06-22

**Authors:** E. Srinivasan, Siranjeevi Nagaraj, Sakthi Rajendran, R. Rajasekaran

**Affiliations:** ^1^Molecular Biophysics Unit, Indian Institute of Science, Bengaluru, India; ^2^Laboratory of Histology, Neuropathology and Neuroanatomy, Faculty of Medicine, ULB Neuroscience Institute, Université Libre de Bruxelles, Brussels, Belgium; ^3^The Steve and Cindy Rasmussen Institute for Genomic Medicine, Nationwide Children's Hospital, Columbus, OH, United States; ^4^Quantitative Biology Lab, Department of Integrative Biology, School of Bio Sciences and Technology, Vellore Institute of Technology (Deemed to be University), Vellore, India

**Keywords:** neurological disorder, Alzheimer's disease, docking, mathematical modeling, Parkinson's disease

Alzheimer's disease (AD) is a major cause of dementia and is characterized by cognitive deficits and behavioral abnormalities. Two established neuropathological hallmarks, amyloid β (Aβ) plaques and hyperphosphorylated tau containing neurofibrillary tangles (NFTs), are associated with AD (Nagaraj et al., 2019; Scheltens et al., 2021). Parkinson's disease (PD) is the second most common neurodegenerative disorder, characterized by uncontrolled movements and coordinating difficulties. At the cellular level, it is due to the loss of dopaminergic neurons and α-synuclein aggregation (Bloem et al., 2021; Srinivasan et al., 2021). The computational and multi-omics approach in gathering data and aiding the design for successful experimentation is crucial, particularly for chronic age-related neurodegenerative diseases such as Alzheimer's and Parkinson's disease, where theragnostic options are limited to managing symptoms and improving quality of life (Golriz Khatami et al., 2020; Myszczynska et al., 2020; Nativio et al., 2020). In this Research Topic, four studies have explored different approaches to address the challenges faced in neurodegenerative diseases for developing novel treatments and diagnoses.

Targeting the clearance of Aβ plaques in Alzheimer's disease is a well-established strategy for effective therapies, as evidenced by recent positive results from clinical trials of aducanumab, lecanemab, and donanemab. Following this approach, Hassan et al. investigated the potential of AZD3293 and Solanezumab as therapeutic agents for treating Alzheimer's disease. By employing molecular docking and dynamic simulations, they studied the binding pattern of these drugs with their respective target proteins, BACE1, and mid-region amyloid-beta. The study demonstrated that AZD3293 was a better candidate for treating Alzheimer's disease than Solanezumab, considering the report from the molecular docking exhibiting strong hydrogen bond interactions forming a stable complex. The outcomes from their MD simulations also corroborated with the docking studies indicating effective interaction of AZD3293 on BACE1. However, despite extensive research aimed at identifying effective therapies for Alzheimer's disease, it remains challenging to pinpoint the root cause of the disease origin due to the disease's heterogeneous endophenotype. Therefore, gathering nodes of genes associated with Alzheimer's disease is an interesting approach to identifying the pathway that should be targeted. In line with this, Sun et al. utilized datasets from three GEO databases (GSE122063, GSE15222, and GSE138260) and identified 111 common differential AD genes. Their study revealed that these differential genes were enriched in the neuroactive ligand-receptor interaction, cAMP signaling pathway, and calcium signaling pathway.

Parkinson's disease, like Alzheimer's disease, has a long prodromal period before clinical symptoms manifest. Therefore, disease-modifying treatments should be directed toward the early stage, prior to the significant decline of dopaminergic neurons and the excessive accumulation of aggregates containing the misfolded synaptic protein α-synuclein. To enhance clinical diagnosis, Song et al. employed machine learning techniques in conjunction with Structural MRI (sMRI) data. Their approach involved utilizing four distinct feature selection methods [ReliefF, graph-theory, recursive feature elimination (RFE), and stability selection] to extract relevant features from both gray and white matter regions. Not only did this method successfully identify group differences between the control and Parkinson's disease subjects, but it also pinpointed the most informative brain regions. By showcasing the capabilities of machine learning models, this study highlights their potential as valuable decision support systems in the diagnosis of Parkinson's disease. In terms of therapeutic applications, Yang et al. proposed a theoretical framework for the degradation of α-synuclein aggregates by theoretical approach. Their mathematical model specifically examines the degradation process of α-synuclein through the autophagy-lysosome pathway (ALP), considering the influence of the mTOR pathway. The model comprises three modules that represent aggregation, ALP, and apoptosis, shedding light on the crucial role played by ALP in maintaining tri-stability. The study findings suggest that mTOR-mediated ALP degradation pathways are the primary mechanism for effectively clearing aggregated α-synuclein, underscoring the significance of the intermediate state in tri-stability. These valuable insights hold the potential to inform and guide future therapeutic interventions for Parkinson's disease.

This Research Topic compiles evidence that highlights the diverse approaches encompassing computational biophysics and mathematical network modeling in advancing therapeutics for neurodegenerative disease pathology and progression. Together, these studies showcase the range of strategies being employed to tackle the challenges posed by neurodegenerative diseases. Although these approaches are predominantly computational, additional validation using *in vitro* and *in vivo* models are imperative to assess their appropriateness and precision. These findings nevertheless carry substantial potential for the development of innovative therapies and decision support systems in the field of neurodegenerative diseases.

## Author contributions

All authors listed have made a substantial, direct, and intellectual contribution to the work and approved it for publication.
